# Potential Health Benefits of Piceatannol-Rich Extracts from *Passiflora edulis* f. *flavicarpa* Seeds on Modulating Gene Expressions Related to Fat Metabolism and Muscle Development in C2C12 Myotubes

**DOI:** 10.1021/acsomega.6c03124

**Published:** 2026-10-19

**Authors:** Nrarat Chobsuay, Jukkarin Srivilai, Tammanoon Rungsang, Pennapa Chonpathompikunlert, Sakara Tunsophon

**Affiliations:** † Department of Physiology, Faculty of Medical Science, 59212 Naresuan University, Phitsanulok 65000, Thailand; ‡ Research and Innovation Center in Cosmetic Sciences and Natural Products, Department of Cosmetic Sciences, School of Pharmaceutical Sciences, 567885 University of Phayao, Phayao 56000, Thailand; § Unit of Excellence for Sustainable Innovation in Cosmetics, Natural Products and Pharmaceuticals (SICNP), School of Pharmaceutical Sciences, University of Phayao, Phayao 56000, Thailand; ∥ Biodiversity Research Center (BRC), Research and Development Group for Bio-Industries, 318616 Thailand Institute of Scientific and Technological Research (TISTR), Pathumthani 12120, Thailand; ⊥ Center of Excellence for Innovation in Chemistry, and Brain and Body Health Research Cluster, Naresuan University, Phitsanulok 65000, Thailand

## Abstract

Yellow passion fruits are widely cultivated in many countries. Throughout industrial manufacturing operations, seeds are frequently disposed of as byproducts. Passion fruit seeds are an abundant natural source of piceatannol, which is known for its antioxidant properties and its ability to modulate protein turnover. This study investigated the potential benefits of passion fruit (PF) extract from pulp and juice, as well as piceatannol-rich (PIC) extract from passion fruit seeds, on palmitic acid (PA)-induced myosteatosis-related muscle atrophy in C2C12 myotubes at the transcriptional level. The PIC extract yielded 5.1% (w/w) with a piceatannol concentration of 0.37% (w/w). The result showed that the PIC extract was more effective in free radical scavenging than the piceatannol standard. Additionally, the results demonstrated that PA promoted lipid accumulation while impairing the muscle formation and glucose absorption. However, both PF and PIC extracts demonstrated a significant reduction in PA-induced lipid accumulation, accompanied by suppression of diacylglycerol acyltransferase 1 (*Dgat1*) expression without altering sirtuin 1 (*Sirt1*) and uncoupling protein 3 (*Ucp3*). In addition, both extracts tended to enhance the expression of myosin heavy chain 1 (*Myh1*) and glucose transporter type 4 (GLUT4), while markedly reducing myostatin (*Mstn*) expression, suggesting improvements in muscle development and glucose uptake. Furthermore, the PIC extract suppressed pancreatic lipase activity, which minimizes spikes in postprandial triglycerides in the blood circulation of rats. These findings emphasize the potential health benefits of PF and PIC extracts in enhancing glucose absorption and inhibiting fat synthesis in C2C12 myotubes, suggesting potential to mitigate lipid accumulation-related muscle development. The promising findings from cell culture suggest further *in vivo* investigation for studying their physiological relevance to muscle wasting caused by fat accumulation in obesity.

## Graphical abstract

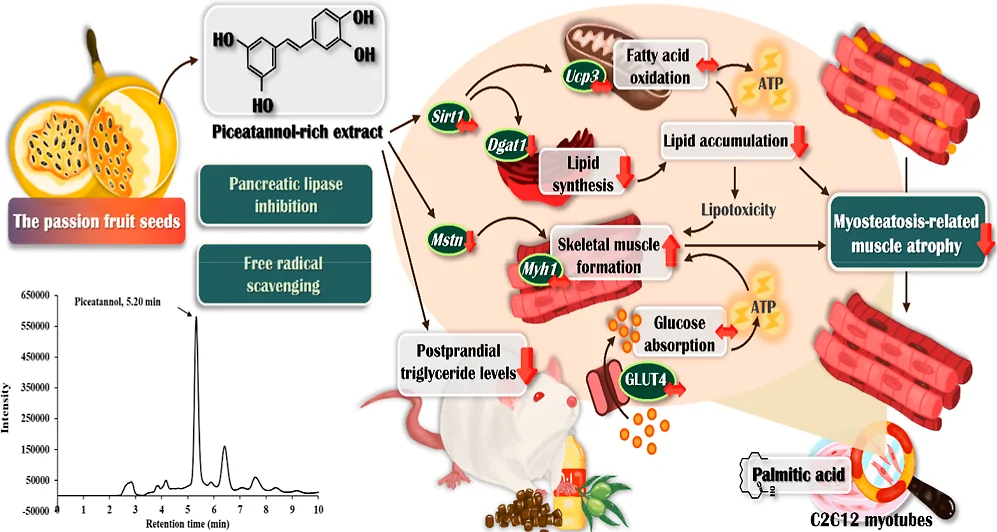

## 1 Introduction

Skeletal muscle is the most abundant tissue, accounting for approximately 40% of the body weight.^1^ It performs numerous essential functions, encompassing the regulation of glucose homeostasis and metabolic rate as well as representing strength, endurance, and performance.^2^ Muscle wasting, also known as atrophy, is characterized by a decrease in muscle size and mass. Muscle atrophy has a prevalence ranging from 24.2 to 40.4% in aging and other clinical diseases in recent decades.^3^ This disorder contributes to a 1–2% decline in muscle mass beyond the age of 50, 1.5% between the ages of 50 and 60, and around 3% per year after that.^4^ Obesity, diabetes, a sedentary lifestyle, aging, and other conditions all contribute to muscle atrophy.^5^ Muscle loss is associated with falls and fractures as well as an increased risk of long-term health problems, including type 2 diabetes, morbidity, and mortality.^6^ Thus, muscle adequacy is critical to long-term health.

Obesity is one of the risk factors for muscular atrophy, a pathological condition distinguished by diminished cross-sectional area (CSA) and reduced muscle mass. Obesity is characterized by increased lipid accumulation in both adipose and nonadipose tissues, such as the liver, pancreas, and skeletal muscles. The balance of fatty acid absorption and oxidation is crucial for lipid storage.^7^ Generally, lipid droplets in the muscle tissue are triglycerides (TG) synthesized from diacylglycerol (DAG) through DGAT1.^8^ During TG formation, lipid intermediates such as DAG and ceramides inhibit the expression of myogenic differentiation 1 (MYOD1), myogenin (MYOG), and mammalian/mechanistic target of rapamycin (MTOR), which are crucial for myoblast proliferation, differentiation, and protein synthesis.^9^ Furthermore, uncoupling proteins (UCPs) play a critical role in metabolic syndrome and obesity. Specifically, UCP3 represents a mitochondrial protein family that participates in the regulation of lipid metabolism and energy homeostasis within skeletal muscle tissues.^10^ Additionally, UCP3 contributes to glucose metabolism by enhancing GLUT4 expression through phosphoinositide 3-kinase (PI3K) activation.^11^ Ectopic fat storage led to mitochondrial dysfunction and cell death due to lipotoxicity.^12^ This problem stimulated the activation of inflammatory cytokines, MSTN-related signaling pathways, insulin resistance, and disrupted protein metabolism pathways, leading to muscle atrophy.^13^ Thus, the regulation of lipid metabolism is critical in myosteatosis-related muscle atrophy. SIRT1 is an NAD^+^-dependent deacetylase that controls lipid metabolism by deacetylating lysine residues in different transcription factors, such as peroxisome proliferative-activated receptor gamma (PPARG) coactivator 1 alpha (PPARGC1A or PGC1α), which plays a key role in mitochondrial biosynthesis and lipid oxidation.^14^ Additionally, the SIRT1-PGC1α axis regulates the genes involved in protein degradation and mitochondrial biogenesis, which was observed to reduce expression in obesity-related muscle wasting.^15^ Thus, activating SIRT1 represents one of the appealing potential pharmacological strategies for preventing muscle atrophy.

Palmitic acid (PA) is a saturated fatty acid that is abundant in blood and is associated with obesity. Excessive PA in the blood is transported to the skeletal muscle, leading to lipid excess and accumulation.^16^ Recent research has found that PA impacts the skeletal muscle in a variety of ways, including myotube formation and hypertrophy, muscle fiber type changes, apoptosis, and interference with insulin signaling pathways and myoprotein metabolism.^17,18^ Additionally, PA is commonly used in the laboratory to mimic aspects of obesity-related muscle atrophy caused by high-fat diets high in saturated fatty acids.^19^ Therefore, the underlying mechanism of PA-induced myosteatosis-related muscle atrophy was investigated in the present study through the expression of genes involved in the lipid metabolism and muscle formation.

Yellow passion fruit (*Passiflora edulis* f. *flavicarpa*) accounts for around 95% of global cultivation. Furthermore, Thailand produces approximately 8,000 tons of passion fruit per year.^20^ Yellow passion fruit contains polyphenols (e.g., quercetin, kaempferol, piceatannol (PIC)) and carotenoids (e.g., β-carotene, cryptoxanthin, zeaxanthin, lutein) that have biological activities such as antioxidant, anti-inflammatory, antihypertensive, hepatoprotective, hypolipidemic, and antidiabetic effects.^21,22^ Previous research has demonstrated antioxidant activity, which helps regulate blood pressure and protect cardiovascular health from the damaging effects of free radicals.^23^ Our previous study reported that the passion fruit extract can inhibit lipase and cholesterol esterase activities.^24^ Additionally, passion fruit extract demonstrated reduced lipid uptake and synthesis, all of which are crucial for lipid digestion, absorption, and accumulation, thereby minimizing lipid presence in the bloodstream and preventing hepatic steatosis.^25^ Furthermore, this fruit is now farmed commercially and is frequently used in the production of juice and soft drinks, resulting in a considerable quantity of waste from the peel and seeds, which account for more than half of the fruit’s weight.^26^ Passion fruit seed contains piceatannol, which exhibits antiobesity, antilipogenic, and antiadipogenic effects.^27^ Additionally, each active ingredient in the passion fruit pulp and seed, such as quercetin, kaempferol, and piceatannol, has shown a protective effect against muscular atrophy through inhibiting muscle ring-finger protein 1 (MuRF1) and F-box protein (Atrogin-1).^28^ From our previous studies, PF extract strongly demonstrated the promising health benefits for antidyslipidemia, antiobesity, and antihepatic steatosis.^25^ The PF extract also showed the ability to regulate parameters related to muscle atrophy, while the PIC extract is an interesting active compound found in passion fruits. However, neither the PF extract nor PIC extract has been investigated for lipid accumulation in myosteatosis-induced muscular atrophy at the postnatal growth stages of muscle development. This is why passion fruit seeds and pulp extracts were used in this investigation to elucidate the potential health effects of natural extracts and gene expression profiling on myosteatosis in relation to muscle development in C2C12 myotubes. Furthermore, the scientific knowledge of PF and PIC extracts may become a valuable element in functional food and dietary supplements to combat obesity-related muscle complications, while also helping minimize waste products from agricultural waste.

## 2 Results

### 2.1 The Validation of the High-Performance Liquid Chromatography Technique for Piceatannol

The calibration curve was shown to be linear between 0.05 and 50 μg/mL concentration range. The correlation coefficients (*R* ^2^) were close to 1, indicating a strong linear correlation at 0.9999. The piceatannol’s limit of detection (LOD) and limit of quantitation (LOQ) were determined as 0.0025 and 0.01 μg/mL, respectively. The HPLC analytical method’s accuracy was verified within the specified concentration range. QC1, QC2, and QC3 were set to concentrations ranging from 5, 20, and 35 μg/mL utilizing the spike approach. The ICH guidelines were utilized to validate compound values, and the method’s precision and accuracy were validated by performing five intraday and three interday analyses. The developed method is suitable for the simultaneous analysis of piceatannol, as evidenced by the high correlation coefficient (>0.9950), % RSD (<5%), and recovery (80–120%) (Table 1).

**Table 1:** Outcomes of the HPLC Method Validation for Piceatannol

validation parameters	value
linearity	0.05–50 μg/mL
correlation coefficient (*R* ^2^)	0.999
linear equation	*y* = 166,452*x* – 19,857
LOD	0.0025 μg/mL
LOQ	0.01 μg/mL
precision (% RSD); intra day	0.25–0.99%
precision (% RSD); inter day	0.52–1.62%
accuracy (% recovery); inter day	100.92–110.50%
analysis time	10 min

### 2.2 Evaluation of Piceatannol in the PIC Extract

After the validation of the HPLC method, the concentration of piceatannol present within the PIC extract was subsequently determined. The PIC extract was detected under optimal conditions: isocratic elution at 1.0 mL/min, detection at 325 nm, and analysis completed in 10 min at ambient temperature. The piceatannol standard exhibited a retention time of 5.20 min, which was consistent with the extract (Figure 1). The amount of piceatannol in the PIC extract was 3.73 ± 0.00 μg/mL or 0.37% w/w.

**Figure 1:**
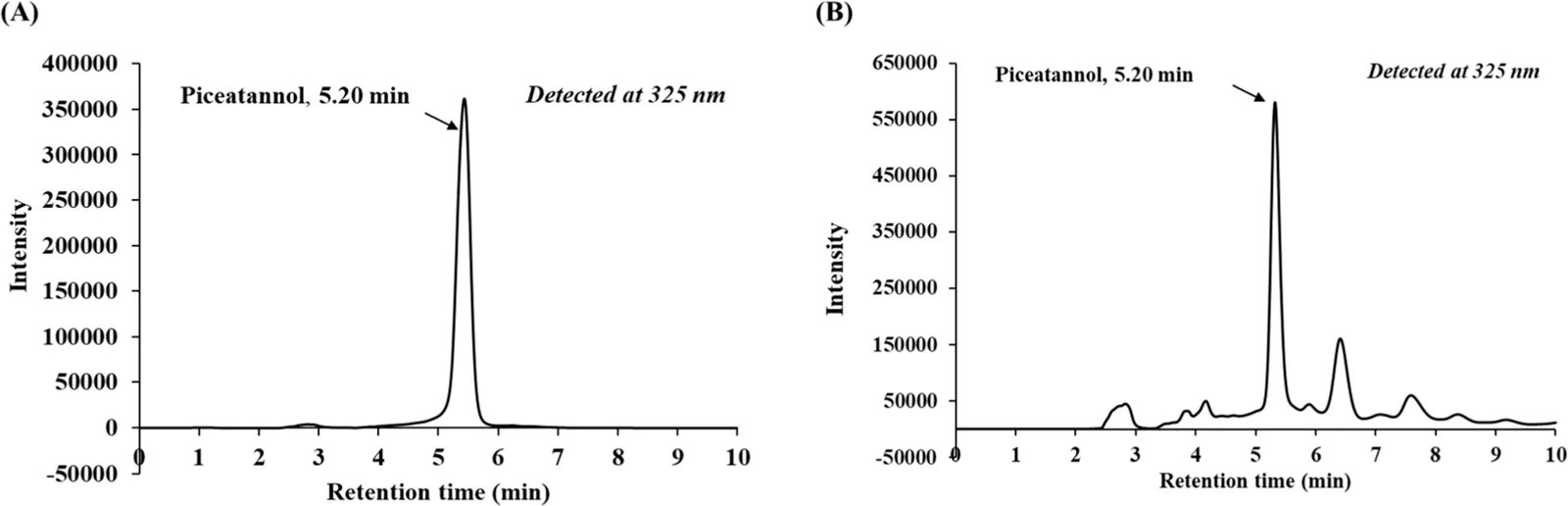
Procedure for obtaining piceatannol from the PIC extract. (A) Piceatannol standard and (B) the PIC extract. PIC extract exhibited a retention time of 5.20 min under detection at 325 nm.

### 2.3 Total Phenolic Content of PIC Extract

The phenolic content was evaluated to establish redox properties of the extract, which is important for antioxidant activity. The linear calibration curve of gallic acid is *y* = 0.0392*x* + 0.0543, with a coefficient of determination (*R* ^2^) value of 0.9996 and provided in gallic acid equivalents (GAE) per gram of dry extract weight (Figure S1). The total phenolic content of PIC extract is 156.39 ± 2.30 mg/g dry weight (Table 2).

**Table 2:** Total Phenolic Content and Antioxidant Activity of PIC Extract^a^

sample	total phenolic content (mgGAE/g extract)	DPPH inhibition IC_50_ (μg/mL or μM)
PIC extract	156.39 ± 2.30	8.70 ± 0.16
piceatannol	-	11.09 ± 1.46, (49.46 ± 6.51 μM)
BHT	-	25.97 ± 0.36, (117.86 ± 1.63 μM)
ascorbic acid	-	8.38 ± 0.10, (47.58 ± 0.57 μM)

a The results were presented as relative mean ± standard deviation (SD) from three independent experiments.

### 2.4 DPPH Scavenging Activity of PIC Extract

The IC_50_ value was used to measure the ability of the PIC extract to scavenge DPPH radicals. Antioxidant concentration is necessary to reduce the DPPH radical by 50%. The antioxidant activity was determined using the DPPH method with ascorbic acid as the standard, and the DPPH radical scavenging ability was 8.38 ± 0.10 μg/mL. Furthermore, the IC_50_ value of butylated hydroxytoluene (BHT) was used as a positive control, which was higher at 25.97 ± 0.36 μg/mL. On the other hand, the IC_50_ values for the piceatannol and PIC extract were 11.09 ± 1.46 and 8.70 ± 0.16 μg/mL, respectively (Table 2). According to these findings, the PIC extract showed stronger antioxidant activity than BHT and piceatannol; however, less than that of ascorbic acid.

### 2.5 The Inhibitory Effect of PIC Extract on Pancreatic Lipase Activity

The ability of the extract to inhibit fat digestion was assessed via its ability to inhibit pancreatic lipase activity, a crucial enzyme for fat digestion. Orlistat was utilized as a positive control for this method due to its ability to completely suppress lipase activity. Orlistat and PIC extract inhibit pancreatic lipase with IC_50_ values of 441.2 and 295.0 μg/mL, respectively (Figure 2). The findings showed that the PIC extract showed potential to inhibit fat digestion compared with orlistat.

**Figure 2:**
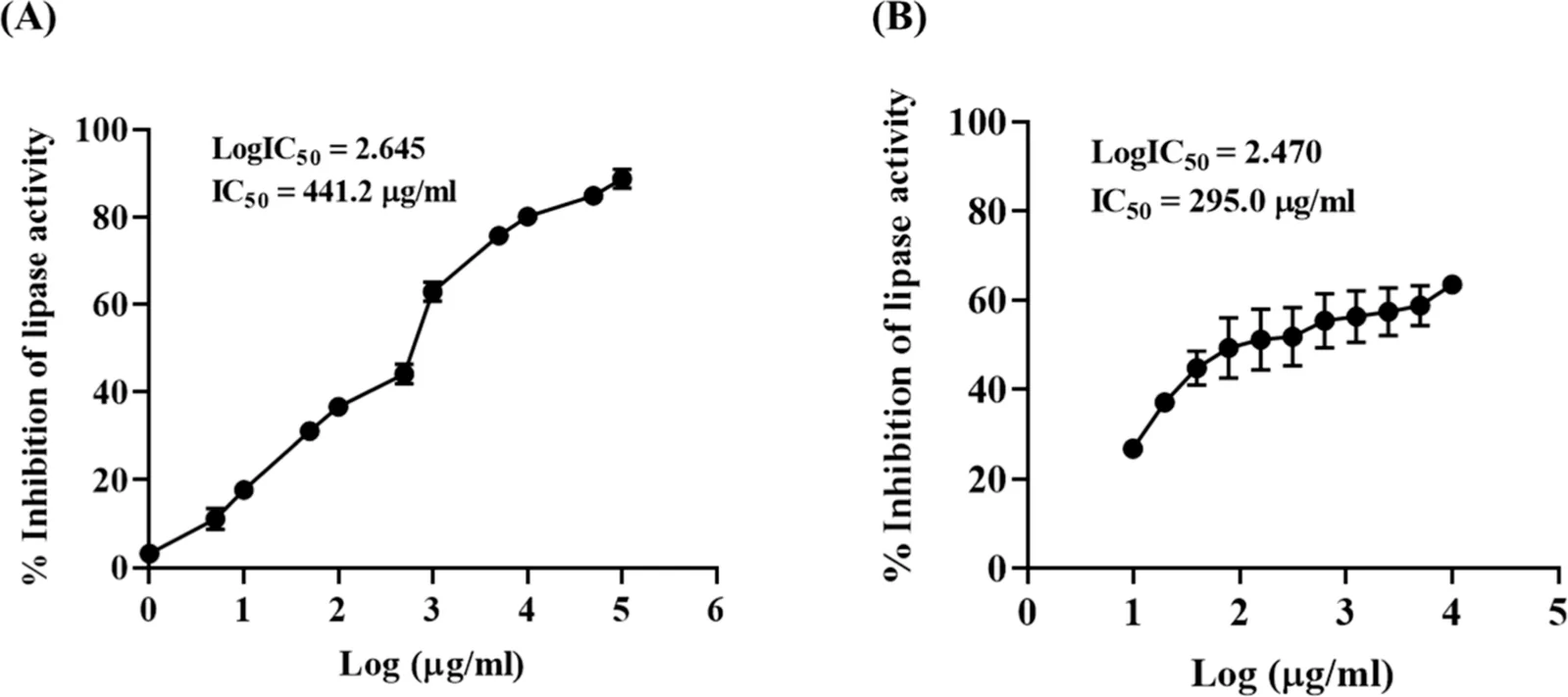
Potential effects of PIC extract on pancreatic lipase inhibition. (A) Orlistat and (B) PIC extract.

### 2.6 The Cytotoxicity and Lipid Accumulation of PA in C2C12 Myotubes

The cytotoxic effects of PA on C2C12 myoblasts were investigated at various concentrations (0.25, 0.50, and 0.75 mM) revealed cell viability of 75.83 ± 1.23, 55.10 ± 3.81, and 44.08 ± 1.42%, respectively. The highest percentage of cell viability was observed at 0.25 mM (Figure S2). The highest level of lipid accumulation was produced at 0.25 mM (180.1 ± 6.31%) (Figure 3).

**Figure 3:**
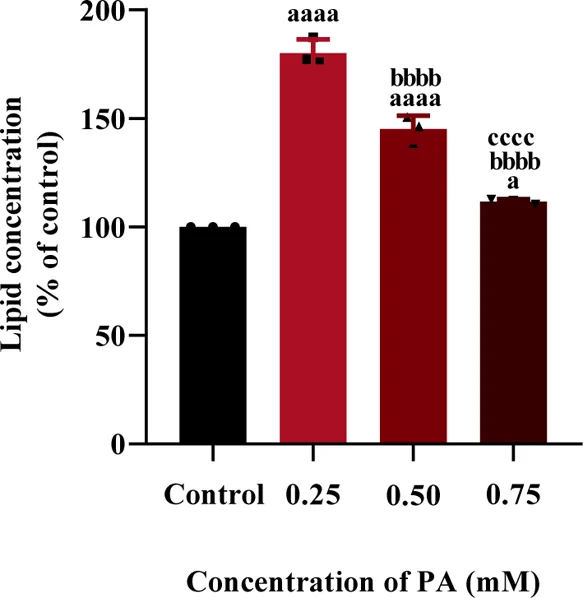
Effects of PA on lipid accumulation in C2C12 myotubes. Quantification of stained lipid droplets was assessed by Oil-Red O staining. The data were reported as mean ± standard deviation (SD), and the tests were repeated three times. Each letter code shows significant differences compared to the control (^a,aaaa^ *p* < 0.05 and 0.0001), 0.25 mM PA (^bbbb^ *p* < 0.0001), and 0.50 mM PA (^cccc^ *p* < 0.0001) based on Tukey’s multiple comparison tests.

### 2.7 The Effect of PF and PIC Extracts on Cell Viability in C2C12 Myotubes

The effect of PF and PIC extracts on cell viability in C2C12 myotubes before and after treatment with PA. PF extract (10, 50, 100, 500, 1000, and 2000 μg/mL) and PIC extract (2.44, 4.88, 12.20, and 24.40 μg/mL) decrease cell viability relative to the control; cell survival was more than 50% (Figure 4A,C). PF and PIC extracts treated with PA exhibited remaining cell survival via inhibited PA toxicity (Figure 4B,D).

**Figure 4:**
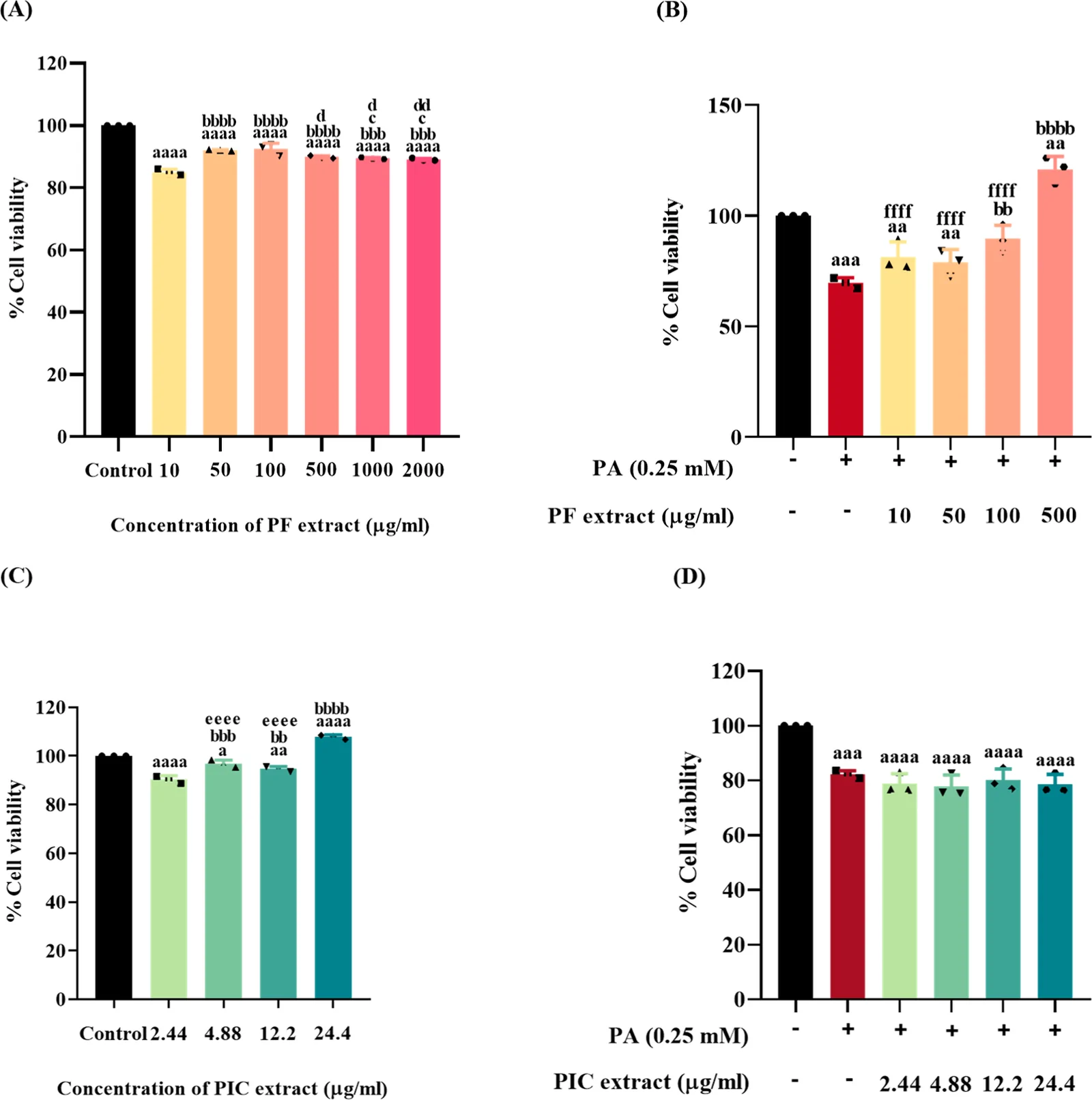
Toxicity of PF or PIC on C2C12 treated with PA. (A) The toxicity of PF extract, (B) the toxicity of PF extract cotreatment with PA, (C) the toxicity of PIC extract, and (D) the toxicity of PIC extract cotreatment with PA. The MTT method was used to measure cell viability. The data were reported as mean ± standard deviation (SD), and the tests were repeated three times. Each letter code shows significant differences compared to the control (^a,aa,aaa,aaaa^ *p* < 0.05, 0.01, 0.001 and 0.0001), 10 μg/mL PF extract or 2.44 μg/mL PIC extract, or 0.25 mM PA (^bb,bbb,bbbb^ *p* < 0.01, 0.001, and 0.0001), 50 μg/mL PF extract (^c^ *p* < 0.05), 100 μg/mL PF extract (^d,dd^ *p* < 0.05 and 0.01), 24.4 μg/mL PIC extract (^eeee^ *p* < 0.0001), and 500 μg/mL PF extract (^ffff^ *p* < 0.0001) based on Tukey’s multiple comparison tests.

### 2.8 The Effect of PF and PIC Extracts on Lipid Accumulation in C2C12 Myotubes

Treatment of C2C12 with 0.25 mM PA, revealed a significant increase in fat formation (123.10 ± 1.99%), and more lipid droplets were stained with Oil-Red O compared to the control group (100.00 ± 0.00%) (Figure 5A). However, treatment with 24.40 μg/mL PIC extract or 500 μg/mL PF extract, demonstrated the potential to inhibit fat storage (109.70 ± 2.72 and 110.00 ± 4.83%, respectively). The PIC extract treated cells at the doses of 24.40 and 12.20 μg/mL (107.40 ± 1.19%) elicited dose-dependent responses when compared to 4.88 μg/mL (121.50 ± 5.34%) (Figure 5B). Furthermore, the mechanism involved in lipid storage indicated that *Sitr1* expression was decreased in the PA group (0.37 ± 0.12) but slightly increased in the 500 μg/mL of PF extract (0.62 ± 0.11) (Figure 5C). The result also indicated that *Ucp3*, which is thought to enhance fatty acid oxidation, was reduced while *Dgat1* expression improved in the PA group (0.26 ± 0.08 and 0.72 ± 0.25, respectively). However, high dosage of PF and PIC extracts exhibited a tendency to increase *Ucp3* expression (0.48 ± 0.03 and 0.47 ± 0.14, respectively). Meanwhile, the *Dgat1* expression was significantly decreased by treatment with 100 and 500 μg/mL PF extracts (0.19 ± 0.07 and 0.12 ± 0.02, respectively), as well as all PIC extract dosages (0.26 ± 0.09, 0.23 ± 0.07, 0.22 ± 0.07, and 0.20 ± 0.03, respectively) (Figure 5D,E). Therefore, our findings suggested that both PF and PIC extracts had the ability to decrease fat accumulation by inhibiting lipid synthesis.

**Figure 5:**
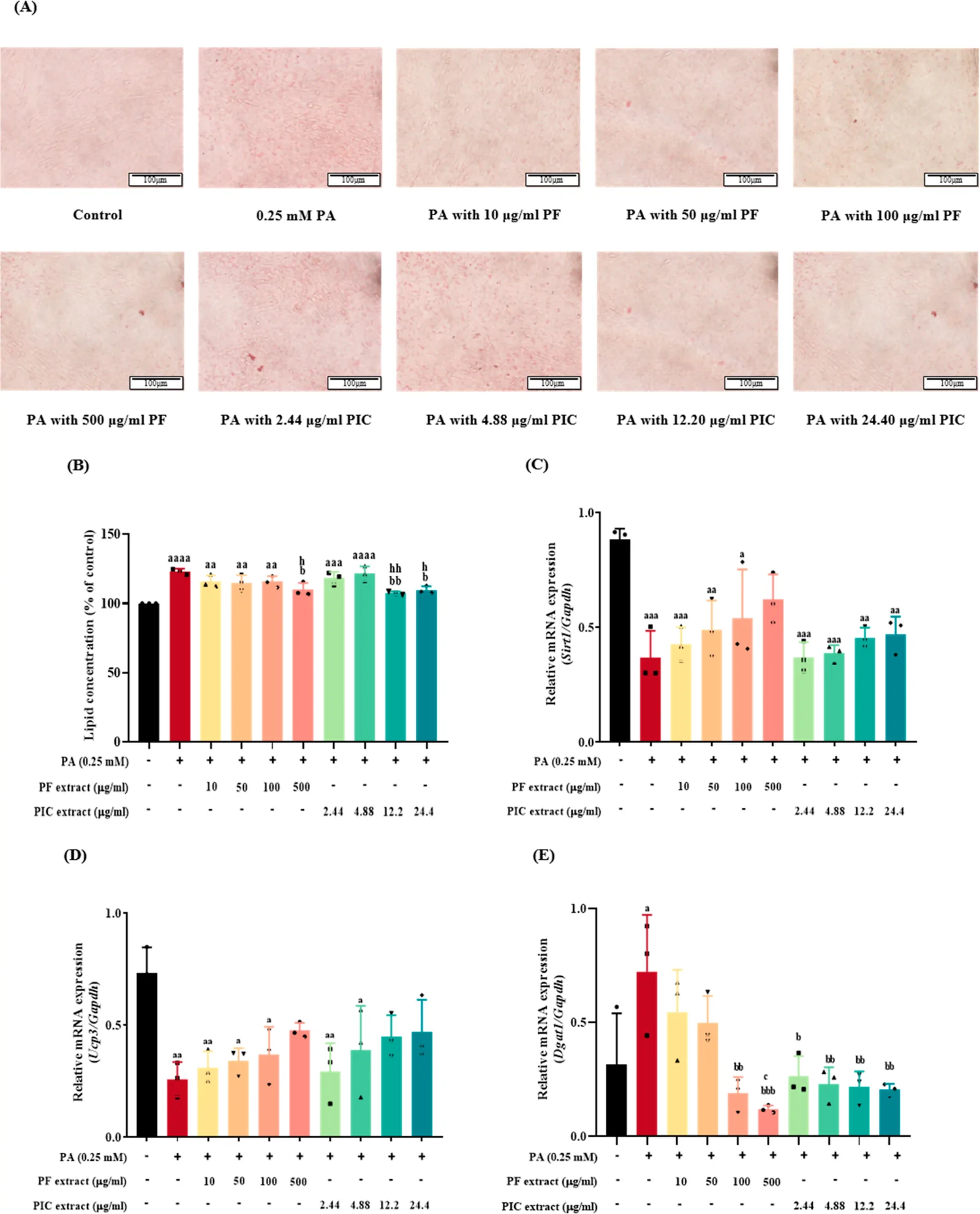
Effects of PF and PIC extracts treated with PA on lipid accumulation in C2C12 myotubes. (A) Lipid storage was stained by oil-red O (scale bar = 100 μm), (B) quantification of stained lipid droplets, the mRNA expressions of *Sitr1* (C), *Ucp3* (D), and *Dgat1* (E) were evaluated using real-time PCR. The data were reported as mean ± SD, and the tests were repeated three times. Each letter code shows significant differences compared to the control (^a,aa,aaa,aaaa^ *p* < 0.05, 0.01, 0.001 and 0.0001), 0.25 mM PA (^b,bb,bbb^ *p* < 0.05, 0.01, and 0.001), 10 μg/mL PF extract (^c^ *p* < 0.05), and 4.88 μg/mL PIC extract (^h,hh^ *p* < 0.05 and 0.01) based on Tukey’s multiple comparison tests.

### 2.9 The Effect of PF and PIC Extracts on Muscle Formation and GLUT4 Expression in C2C12 Myotubes

MSTN is a key regulator of muscle proliferation and differentiation. Our findings indicated that PA enhanced the *Mstn* expression while decreasing *Myh1* expression (0.92 ± 0.06 and 0.32 ± 0.11, respectively), which is a transcript for mature fast-type muscle fibers (Figure 6A,B). Also, GLUT4 expression was lower in the PA group compared to the control group (65.10 ± 7.49 and 126.10 ± 12.14, respectively) (Figure 6C,D). However, PF and PIC extracts showed the ability to enhance muscle growth by slightly increasing *Myh1* (0.67 ± 0.16 and 0.52 ± 0.20, respectively), while also reducing *Mstn* expression in a dose-dependent manner. Moreover, 100 μg/mL PIC extract showed a tendency to increase GLUT4 expression (88.67 ± 7.72); meanwhile, 500 μg/mL PF extract did not show a difference from the control group (95.47 ± 17.99). These findings demonstrated that PF and PIC extracts improved muscle development and tended to improve GLUT4 expression.

**Figure 6:**
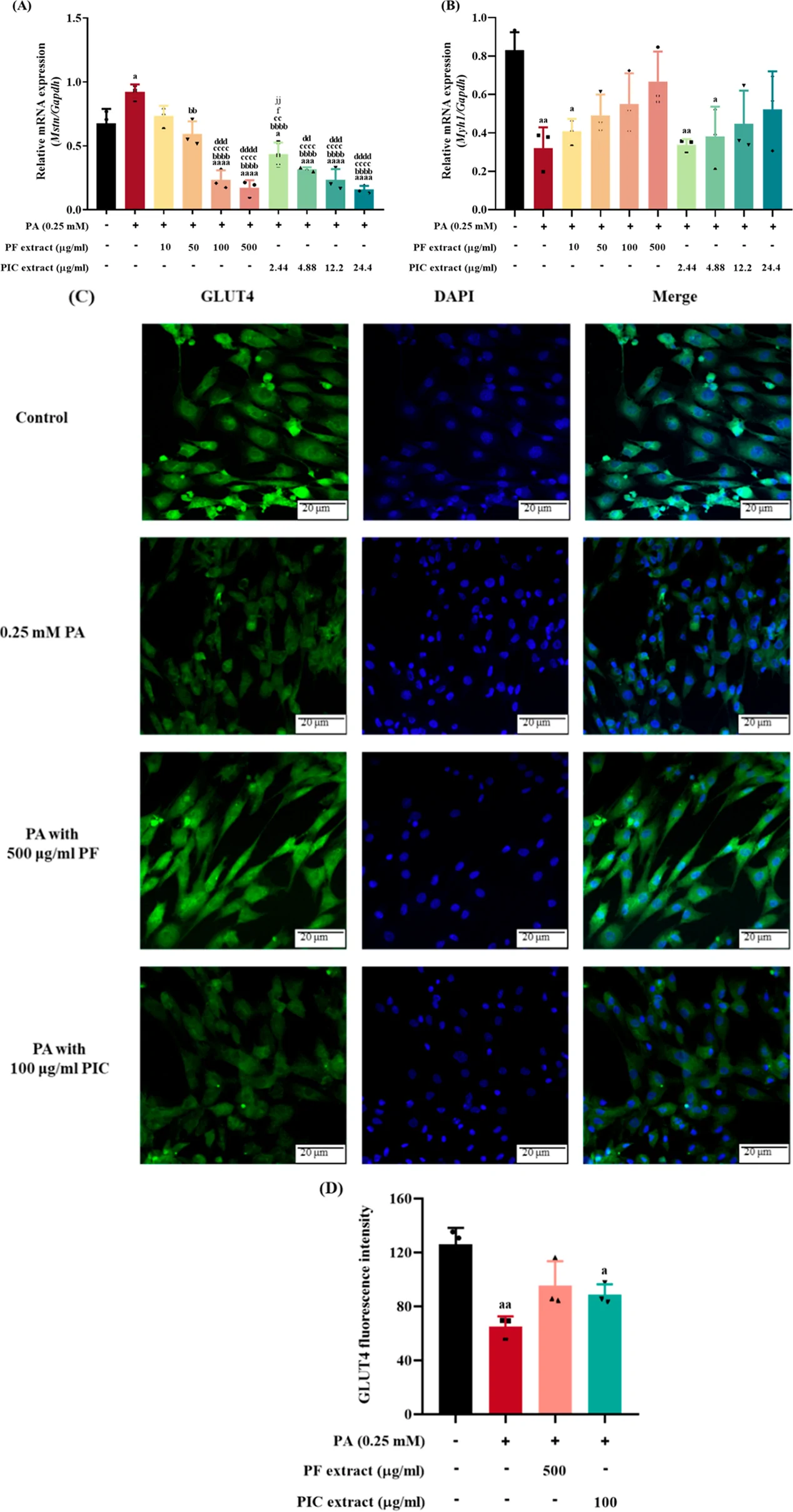
Effects of PF and PIC extracts treated with PA on muscle formation and GLUT4 expression in C2C12 myotubes. The mRNA expressions of *Mstn* (A) and *Myh1* (B) were evaluated using real-time PCR, (C) GLUT4 expression was immunostained using Glut4 antibodies and DAPI, merged, and visualized using confocal microscopy (scale bar = 20 μm), and (D) total GLUT4 expression was determined and considered as total fluorescence intensity. The data were reported as mean ± standard deviation (SD), and the tests were repeated three times. Each letter code shows significant differences compared to the control (^a,aa,aaa,aaaa^ *p* < 0.05, 0.01, 0.001, and 0.0001), 0.25 mM PA (^bb,bbbb^ *p* < 0.01 and 0.0001), 10 μg/mL PF extract (^cc,cccc^ *p* < 0.01 and 0.0001), 50 μg/mL PF extract (^dd,ddd,dddd^ *p* < 0.01, 0.001, and 0.0001), 500 μg/mL PF extract (^f^ *p* < 0.05), and 24.4 μg/mL PIC extract (^jj^ *p* < 0.01) based on Tukey’s multiple comparison tests.

### 2.10 PF and PIC Extracts Mitigated Postprandial Triglyceride Levels in Obese Rats

According to an *in vitro* study, the PF and PIC extracts have the ability to inhibit lipase activity, which may facilitate the prevention of dietary fat absorption and the subsequent elimination of lipids following a high-fat meal. Thus, the rats were administered PF or PIC extracts to assess the ability of both extracts to eliminate fat. Our findings showed that the obese group showed a higher tendency for TG levels than the control group at 4 h (213.30 ± 77.66 and 144.30 ± 15.37, respectively) (Figure 7A). In contrast to the obese group, the TG levels in the blood showed significantly decreases in the PF at 4 h, whereas PIC reduced TG at 2 and 4 h. The PIC extract group had a significantly lower area under the curve (AUC) values than the obese group (73.42 ± 4.74 and 398.90 ± 241.30, respectively), while the PF group had a trend to drop values (116.20 ± 31.60) but was not significantly different compared to the obese group (Figure 7B). Although both extracts were able to prevent TG levels from spiking after fat administration, the PIC extract showed a more effective decrease in total TG exposure in the circulation during the oral fat tolerance test (OFTT).

**Figure 7:**
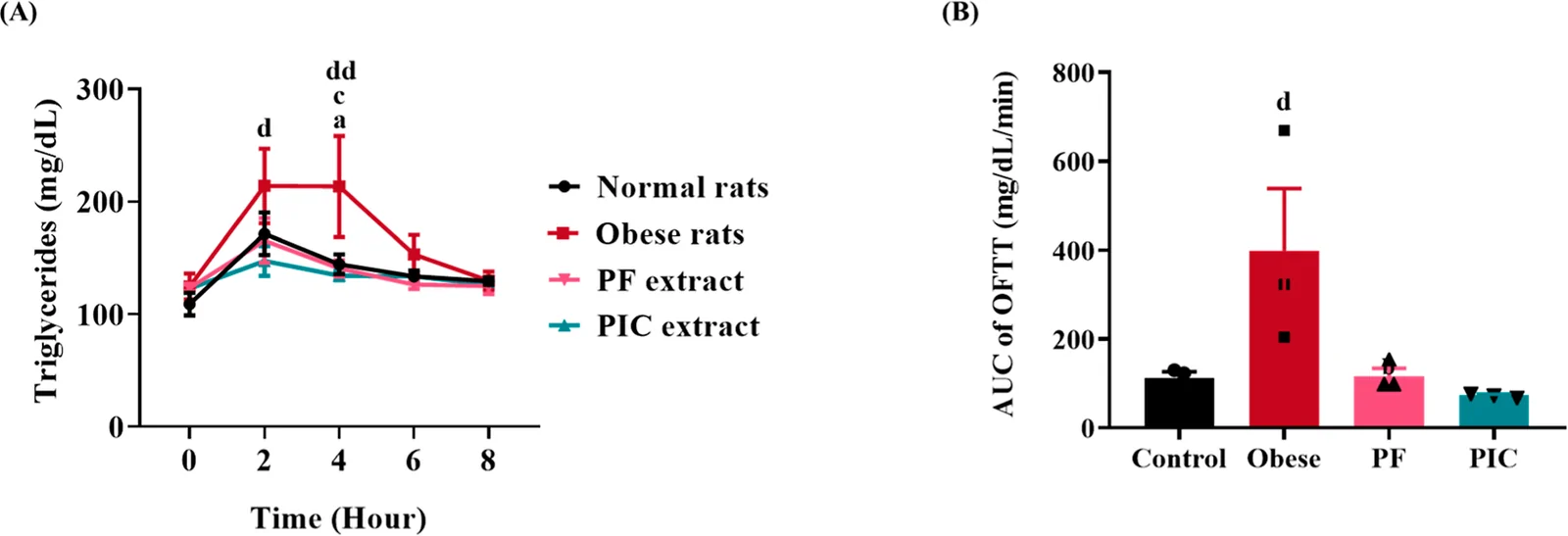
Effects of PF and PIC extracts on postprandial triglyceride levels in obese rats. (A) Plasma TG levels and (B) AUC values during the OFTT. The data were reported as mean ± standard deviation (SD) (*n* = 3). Each letter code shows significant differenced compared to the control group (^a^ *p* < 0.05), 500 mg/kg PF extract group (^c^ *p* < 0.05), and 45 mg/kg PIC extract group (^d,dd^ *p* < 0.05 and 0.01) based on Tukey’s multiple comparison tests, and two-way ANOVA (repeated-measures ANOVA) was performed for the oral fat tolerance tests (OFTT).

## 3 Discussion

The present study demonstrated that the seeds of passion fruit extract contained piceatannol, which is consistent with an earlier study that found piceatannol in the seeds of passion fruit, as well as other fruits such as grapes, teas, berries, and so on.^29^ The amount of piceatannol in the PIC extract in the present study was found to be 0.37% (w/w), which is less than the previous reported value of 3.68% (w/w),^30^ or nearly 10 times. These findings depend on the cultivar and extraction method.^30^ In addition, our previous research also reported that the pulp of yellow passion fruit contains other bioactive compounds, such as β-carotene and γ-tocopherol.^24^ Additionally, pancreatic lipase is an important enzyme that digests triglycerides into free fatty acids and monoglycerides, which are essential for lipid absorption and metabolism.^31^ Consequently, this enzyme is of interest as a target for obesity treatment. Our previous and present results showed that both extracts suppressed enzyme-mediated digestion of fats and exhibited a potent radical scavenging activity.^24^ These findings showed that the PIC and PF extracts have the ability to inhibit the enzyme involved in fat digestion and absorption, which may prevent fat accumulation and its consequences.

Muscle atrophy comes from muscle loss and dysfunction, a condition frequently correlated with obesity. PA is a common saturated fatty acid used to promote ectopic fat accumulation. Obesity has also been associated with high levels of palmitic acid.^32,33^ Then, palmitic acid is commonly used to mimic obesity, metabolic syndrome, and lipid accumulation in an *in vitro* study.^34,35^ In this study, we used PA-induced fat formation in C2C12 cells and found that 0.25 mM PA increased lipid accumulation with mild toxicity or stress in cells. However, the cell viability is more than 70%, which is classified as noncytotoxic according to ISO 10993-5-2009.^36^ On the other hand, 0.50 and 0.75 mM PA were observed to have less cell viability, indicating cytotoxicity in high doses of PA. Lipotoxicity impairs the endoplasmic reticulum stress response, increases oxidative damage, and accumulates lipid intermediates, including diacylglycerol (DAG) and ceramides, which can eventually cause cell death.^37^ Our findings were similar to a prior study, which showed that increasing the PA dosage from 0.75 to 1 mM promoted cell death by 25–60%.^38^ These findings indicate that PA increased fat deposition, thus having a negative effect on cell survival. However, PF and PIC extracts demonstrated that they were not harmful to cells and enhanced cell survival in PA-induced muscle atrophy. Additionally, the SIRT1-PGC1α axis regulates lipid oxidation, whereas DGAT1 controls TG synthesis. Our findings demonstrated that PA promoted lipid accumulation (23% increase over control) by stimulating *Dgat1* expression while inhibiting *Sirt1* and *Ucp3* expressions, leading to fat formation and accumulation. These findings are consistent with a previous study, which suggested that PA enhanced DGAT1 expression while simultaneously increasing TG levels by approximately 20% over control.^39^ Another study also found that PA is a negative regulator of SIRT1 and PPARGC1A expression, which is associated with UCP3 expression.^40,41^ Although *Sirt1* and *Ucp3* expressions demonstrated a tendency toward upregulation when exposed to PF extract or PIC extract, a concurrent reduction in *Dgat1* expression and diminished fat accumulation of 14 and 13% were documented at concentrations of 24.4 μg/mL PIC extract and 500 μg/mL PF extract, respectively. This suggests that *Sirt1* and *Ucp3*-mediated lipid accumulation may not play a primary role in this consequence. Previous reports found that 100 μM piceatannol has the potential to reduce lipid accumulation by 20% via inhibiting fatty acid absorption and lipogenesis while increasing fatty acid oxidation.^42^ Thus, our findings contradict previous research on the PIC extract’s ability to decrease lipogenesis without affecting fat metabolism. Passion fruit pulp contains phenolic compounds, which have been shown to decrease the accumulation of lipids in adipose tissues, hence preventing obesity-related metabolic disorders.^43^ Additionally, both extracts also reduced postprandial triglyceride levels that is the temporary rise in triglycerides in the bloodstream after a meal and are associated with fat digestion and absorption.^44^ These results are consistent with our previous studies that showed PIC and PF extracts can inhibit pancreatic lipase activity and the excretion of triglyceride in feces.^24,25^ Thus, the pulp and seed of passion fruit extract demonstrated potential in preventing dyslipidemia and fat storage, and it might help prevent other fat-related conditions.

Furthermore, the negative consequences of lipid accumulation and uptake such as muscle development were investigated. Our findings show that PA promotes *Mstn* expression (up 28.57% from control), a muscle formation inhibitor, while decreasing *Myh1* expression (down 62.5% from control), which is transcribed for mature muscle fibers. Consistent with previous findings, PA inhibited myotube formation by upregulating MSTN expression, resulting in reduced myotube area and myogenic index, as well as downregulating MHCIIx gene expression by around 40%, such as MYH1, a fast-twitch fiber isoform, which is termed myotube atrophy.^18,45^ However, PF and PIC extracts demonstrated comparative effectiveness in minimizing *Mstn* expression (85.71% of both extracts) and could moderately enhance *Myh1* expression (113 and 73% in PF and PIC extracts, respectively). The role of fast-twitch fibers in the prevention of muscle wasting and therefore maintaining functional strength is because they play an important role in the maintenance of overall health and strength.^46^ These findings suggest that both PF and PIC extracts have the ability to prevent myotube atrophy by enhancing the myotube formation. Additionally, muscular atrophy is also caused by disruptions in GLUT4 expression, which controls glucose absorption, insulin resistance, and muscular function.^47^ As previously stated, PA suppressed *Ucp3* expression while increasing *Mstn* expression, which disrupts the PI3K pathway, thus inhibiting GLUT4 translocation to the cell surface, resulting in impaired muscle glucose absorption and function.^11^ However, PF and PIC extracts were shown to slightly upregulate *Ucp3* expression while suppressing *Mstn* expression, resulting in a tendency to improve the level of GLUT4 expression (46.65 and 36.21% in PF and PIC extracts, respectively). Furthermore, a prior study demonstrated that piceatannol, a substance present in passion fruit, enhanced GLUT4 mRNA expression by 100% over the control group.^48^ Improved GLUT4 regulates cellular glucose transport and affects the metabolic health and function of skeletal muscle. The findings indicate that PF and PIC extracts have an equivalent ability to improve glucose absorption by elevating GLUT4 expression. Additionally, PA might trigger downstream changes in gene expression and cellular phenotype by influencing the expression of genes involved in metabolism and muscle fiber type.^49^ However, this study demonstrated the potential health benefits of PF and PIC extracts and warrants further investigation since protein metabolism and metabolic health are critical for controlling muscle atrophy. The limitation of this study is that the dosage of palmitic acid was selected based only on fat accumulation and previous research, without conducting lower dosages of PA and gene expression assessment before proceeding with subsequent experiments. Second, only the effects of both extracts on lipid accumulation and muscle development in C2C12 were determined. The alteration in gene expression and cellular phenotype may have been influenced by the combination of lipid accumulation and lipotoxic stress, which is a limitation of the present model. Furthermore, the relatively small number of biological replicates (*n* = 3), despite technical triplicates, represents a limitation of the present study and may contribute to the observed variability in the gene expression. Future studies *in vivo* in terms of oxidative stress, apoptosis, and inflammation, which are involved in protein homeostasis in muscle atrophy-associated obesity, are necessary to validate the underlying mechanism in physiological relevance.

## 4 Conclusion

In summary, the seed extract was found to contain piceatannol, while both the seed and passion fruit extracts demonstrate promising antioxidant and antiobesity characteristics. Furthermore, PA was used in inducing lipid accumulation-related muscle atrophy models through altering the balance of fat metabolism and limiting muscle development. The PF and PIC extracts demonstrated the potential to counteract the effects of PA by activating genes involved in fat oxidation while modulating genes involved in lipid synthesis and negative regulators of muscle development. Interestingly, the PIC extract was found to minimize total TG exposure in the circulation after a high-fat diet. These findings provide new insights into possible treatment targets for combating this health problem. However, further investigation into other potential antiatrophy signaling pathways and *in vivo* efficacies for both extracts is important.

## 5 Materials and Methods

### 5.1 PF and PIC Extracts Preparation

*P. edulis* f. *flavicarpa* O. Deg. (BK. 082283) or yellow passion fruit was collected from Chiang Mai, Thailand. The 4500 g passion fruit pulp and juice (excluding the seeds) were extracted according to the previous study,^24^ yielding 545.4 g of PF extract (12.12% w/w). For PIC extract, it was prepared and optimized using a previously published technique,^50^ with slight modifications. Seeds were first washed with water to remove impurities, then dried in the airflow. Dried seeds were ground and sieved using a 300 μm mesh to eliminate fine wax particles. Seed coat particles larger than 300 μm were collected and washed in distilled water until they were clear, removing the remaining fat and wax. After that, the cleaned seed coat powder was dried at 50 ± 0.5 °C for 48 h. Then, the dried seed coat material (700 g) was extracted with 75% ethanol using ultrasonic-assisted extraction at a frequency of 40 kHz at 45 °C for 30 min. The extraction procedure was repeated three times under the same conditions. The black powdered extract of passion fruit seeds was obtained after being filtered and concentrated under reduced pressure using a rotary evaporator and dried on a boiling water bath under gentle heating conditions. The yield of PIC extract was 35.7 g or 5.1% w/w.

### 5.2 High-Performance Liquid Chromatography (HPLC) Analysis

The analysis of piceatannol was performed using a Shimadzu Prominence UFLC system equipped with an LC-2050C pump, SPD-20A UV–vis detector (230 V), Phenomenex C18(2) column (5 μm, 250 mm × 4.6 mm) along with a guard column (uHPLC C18, 5 μm, 20 mm × 4.5 mm). Piceatannol standard (98% purity, LGC standards GmbH, Germany) and PIC extract were injected 20 μL for each run and analyzed under isocratic conditions at a flow rate of 1.0 mL/min and detection at 325 nm at ambient temperature. The mobile phase consisted of 1.0% (v/v) formic acid in deionized water (A) and acetonitrile (B) at a 65:35 ratio, respectively.

### 5.3 Method Validation

The HPLC technique was validated for linearity and range, limit of detection (LOD), limit of quantification (LOQ), accuracy (% recovery), and precision (% RSD) to ensure the reliability and accuracy of the analysis in accordance with the International Council for Harmonization of Technical Requirements for Pharmaceuticals for Human Use (ICH) guidelines.^51^ The piceatannol stock solution was prepared in 99% ethanol (HPLC grade, Labscan, Bangkok) and a calibration curve was constructed within the concentration range of 0.05–50 μg/mL.

### 5.4 Determination of Total Phenolic Content

The total phenolic content was determined by the total phenolic content method.^52^ The extract (20 μL) was mixed with 100 μL of 10% Folin–Ciocalteu reagent. After that, 10% Na_2_CO_3_ (80 μL) was added and incubated at room temperature for 30 min. The absorbance was measured at 765 nm using a microplate reader (BioTek Instruments, Winooski, VT), and the phenolic content of the extract was expressed as gallic acid equivalent (GAE) in milligrams per gram of extract sample.

### 5.5 Determination of Antioxidant Activity

The 2,2-diphenyl-1-picrylhydrazyl (DPPH) radical-scavenging activity was modified from previous research.^53^ 100 μL of extract was mixed with 100 μL of 0.5 mM DPPH solution. The solution was incubated at room temperature for 30 min. The absorbance was measured at 517 nm using a UV–vis spectrophotometer (DU 800; Beckman Coulter, Fullerton, CA). The percentage of DPPH inhibition was calculated using the following equation: % DPPH inhibition = 100 – ((absorbance of extract – absorbance of blank/absorbance of control) × 100).

### 5.6 The Pancreatic Lipase Inhibition Assay

A previous study described the pancreatic lipase inhibition assay.^54^ The reaction consists of pancreatic lipase, 4-nitrophenyl laurate, and PIC extract or orlistat. The solution was then mixed and incubated for 2 h at 37 °C. Finally, the solution was measured at 400 nm using a microplate reader (BioTek Instruments, Winooski, VT).

### 5.7 Cell Culture

C2C12 myoblasts (ATCC CRL-1772) were cultivated in Dulbecco’s Modified Eagle’s Medium (DMEM) with high glucose concentration, supplemented with 10% fetal bovine serum (FBS), 1% antibiotic (penicillin and streptomycin), and maintained in a humidified 5% CO_2_ atmosphere at 37 °C. Cells were cultured with 10% FBS in DMEM, and when the cells reached 60–70% confluence, they were treated with 2% FBS in DMEM to stimulate myotube differentiation.

### 5.8 Cytotoxicity Assays

The effect of PIC and PF extracts on the cytotoxicity of C2C12 myoblasts was evaluated after 24 h of treatment with PIC extract at 2.44–24.4 μg/mL (piceatannol equivalent of 10–100 μM) and PF extract (5, 10, 50, 100, 500, 1000, and 2000 μg/mL).^24,55^ Twenty microliters of 3-(4,5-dimethylthiazol-2-yl)- 2,5-diphenyltetrazolium bromide (MTT) solution was added to each well and incubated at 37 °C in a CO_2_ incubator. The solution was discarded and replaced with dimethyl sulfoxide (DMSO) (150 μL) in each well, and then the absorbance was measured at 540 nm using a microplate reader. The percentage of cell viability was determined by comparing with the control.

### 5.9 Treatment in the C2C12 Cell Culture

In the myotube differentiation process, PIC and PF extracts were treated for 24 h with 0.25 mM PA on day 3 of the differentiation process. PA conjugated with bovine serum albumin (BSA)-fatty acid-free was used to induce an atrophy model using a previously reported method.^56^

### 5.10 Oil-Red O Staining

C2C12 cells were rinsed with PBS and fixed with 4% formaldehyde for 30 min. The cells were washed with 60% isopropanol and incubated for 30 min with 0.5% Oil-red O dissolved in 100% isopropanol. Oil-red O was washed with PBS and dissolved in 100% isopropanol. The absorbance was measured at 510 nm.^57^

### 5.11 Real-Time Polymerase Chain Reaction

The procedure was modified from our previous study.^58^ Total cellular RNA was extracted from the cells using Trizol reagents (Molecular Research Centre, Inc.). cDNA was prepared using ReverTra Ace qPCR RT Master Mix with gDNA Remover. A THUNDERBIRD Next SYBR qPCR Mix was used for real-time PCR analysis using a Pharmaceutical Analytics QuantStudio 5 Real-Time PCR system. The mRNA expressions of *Sirt1*, *Ucp3*, *Dgat1*, *Myh1*, and *Mstn* were assessed by using glyceraldehyde-3-phosphate dehydrogenase (*Gapdh*) as the reference gene. The mRNA levels were expressed as mean expression. The primers used are listed in Table S1.

### 5.12 Immunofluorescence

The method was based on a previous study.^59^ C2C12 cells were placed on 6-well plates with cover glasses. Cell proliferation and differentiation were observed. The cells were fixed with 4% formaldehyde for 30 min following a 24 h treatment with both extracts. Cells were then incubated for 5 min with 0.1% Triton-X and for 1 h with 1% BSA. Cells were blocked with the primary GLUT4 antibody (1:1000, PA5-23052) in buffer (1% BSA in PBS) overnight at 4 °C before being incubated with the FITC-conjugated secondary antibody (1:1000, F2765) for 1 h at room temperature. After that, cells were incubated with DAPI and Hoechst (HI1399) for 10 min. Finally, the GLUT4 expression was observed under a confocal microscope (Zeiss LSM 900 with Airyscan).

### 5.13 Postprandial Triglyceride Responses in Rats

Animals were handled in accordance with the guidelines established by the Institutional Animal Care Committee of Naresuan University, Phitsanulok, Thailand. Five-week-old male Sprague-Dawley rats (NU-AE 621023) weighing 120–150 g were purchased from Nomura Siam International Co. Ltd. in Bangkok, Thailand. The rats were placed in cages with a regulated temperature (22 °C) and humidity (55 ± 10%) and were subjected to a 12 h light and 12 h dark cycle. Throughout a week-long acclimatization period, they were granted unrestricted access to food and water. After acclimatization, the rats were randomly divided into four experimental groups (*n* = 3). Rats were induced to be obese with a high-fat diet for 5 weeks before being evaluated for fat tolerance with OFTT. The baseline lipid level was assessed before orally administering olive oil or olive oil with 45 mg/kg of PIC or 500 mg/kg of PF extracts.^25,60^ Blood was collected from the tail veins before and 1–8 h after the administration. Triglyceride levels in the serum were measured using a lipid strip and Accutrend Plus meter (Roche Diagnostics Deutschland GmbH, Germany).

### 5.14 Statistical Analysis

GraphPad Prism 8 (GraphPad Software Inc., San Diego, CA) was used to generate the graphs and analyze all of the data using one-way ANOVA followed by Tukey’s multiple comparison tests, and the findings were provided as the SD. Additionally, two-way ANOVA (repeated-measures ANOVA) was performed for OFTT analysis. A statistically significant difference was determined at *p* < 0.05.

## Supplementary Material



## Data Availability

The data sets are available from the corresponding author on reasonable request.

## Notes

Gene sequencing, standard curve for total phenolic content, PA cytotoxicity in C2C12 cell culture, and the melt curves for each analyzed gene (PDF)

## Abbreviations

DGAT1: diacylglycerol acyltransferase 1
GLUT4: glucose transporter type 4
MSTN: myostatin
MTOR: mammalian/mechanistic target of rapamycin
MYH1: myosin heavy chain 1
OFTT: oral fat tolerance tests
PA: palmitic acid
PI3K: phosphoinositide 3-kinase
TG: triglycerides
SIRT1: sirtuin 1
UCP3: uncoupling protein 3
