# Supplementary material for: Potential Health Benefits of Piceatannol-Rich Extracts from Passiflora edulis f. flavicarpa Seeds on Modulating Gene Expressions Related to Fat Metabolism and Muscle Development in C2C12 Myotubes
Source: ACS Omega. 2026 Oct 19;11(43):66127–38. doi: 10.1021/acsomega.6c03124 (PMC13737894; doi:10.1021/acsomega.6c03124)
Supplement: Supplementary file 1 [file ao6c03124_si_001.pdf]

Supplementary Information

Potential health benefits of piceatannol-rich extract from *Passiflora edulis* f. *flavicarpa* seeds on modulating gene expressions related to fat metabolism and muscle development in C2C12 myotubes

Nrarat Chobsuay[1], Jukkarin Srivilai[2,3], Tammanoon Rungsang[2,3], Pennapa Chonpathompikunlert[4], and Sakara Tunsophon[1,5,*]

[1] *Department of Physiology, Faculty of Medical Science, Naresuan University, Phitsanulok 65000, Thailand*

[2] *Research and Innovation Center in Cosmetic Sciences and Natural Products, Department of Cosmetic Sciences, School of Pharmaceutical Sciences, University of Phayao, Phayao 56000, Thailand*

[3] *Unit of Excellence for Sustainable Innovation in Cosmetics, Natural Products and Pharmaceuticals (SICNP), School of Pharmaceutical Sciences, University of Phayao, Phayao 56000, Thailand*

[4] *Biodiversity Research Center (BRC), Research and Development Group for Bio-Industries, Thailand Institute of Scientific and Technological Research (TISTR), Pathumthani 12120, Thailand*

[5] *Center of Excellence for Innovation in Chemistry, and Brain and Body Health Research Cluster, Naresuan University, Phitsanulok 65000, Thailand*

([*]Corresponding author's e-mail: sakarat@nu.ac.th)

Figure S1. Gallic acid standard curve for total phenolic content

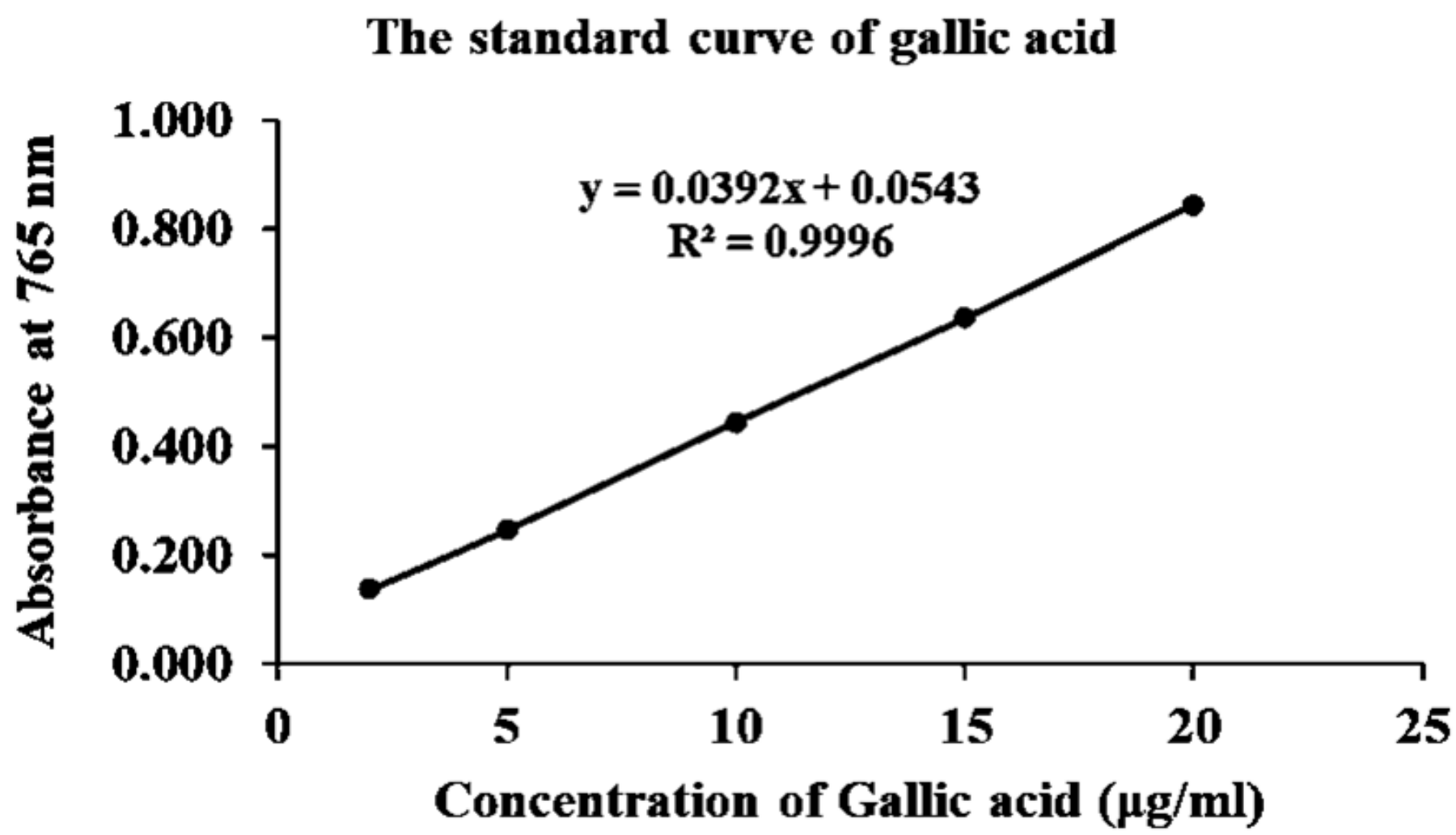

Figure S2. The cytotoxicity of PA to C2C12 cell viability.

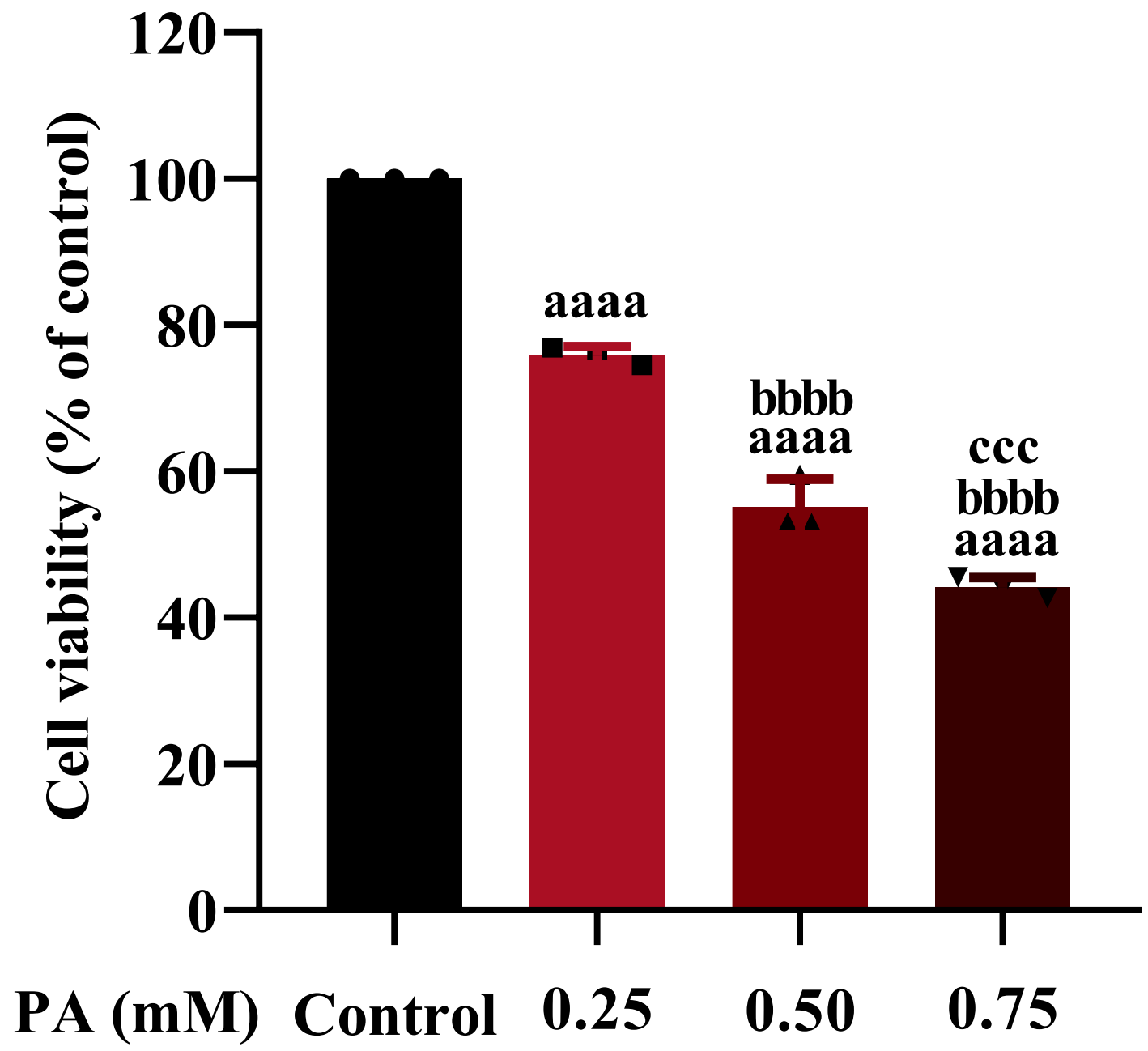

Figure S2. Effects of PA on cell viability in C2C12 myotubes. The data were reported as mean ± standard deviation (SD), and the tests were repeated 3 times. Each letter code shows significant differences compared to the control ([aaaa] $p < 0.0001$), 0.25 mM PA ([bbbb] $p < 0.0001$), and 0.50 mM PA ([ccc] $p < 0.001$) based on Tukey's multiple comparison tests.

Figure S3. The melt curves of *Dgat1*

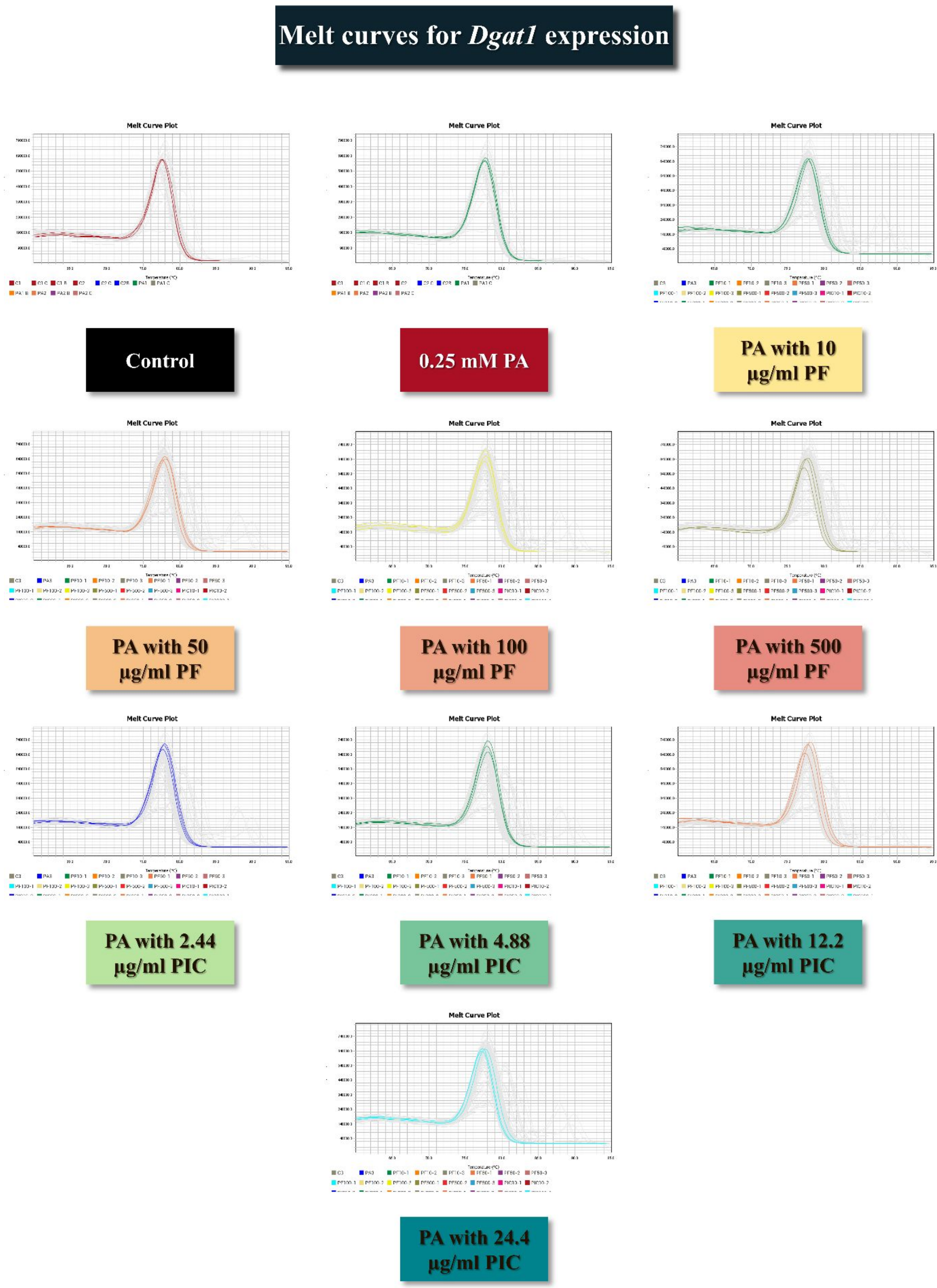

Figure S4. The melt curves of *Mstn*

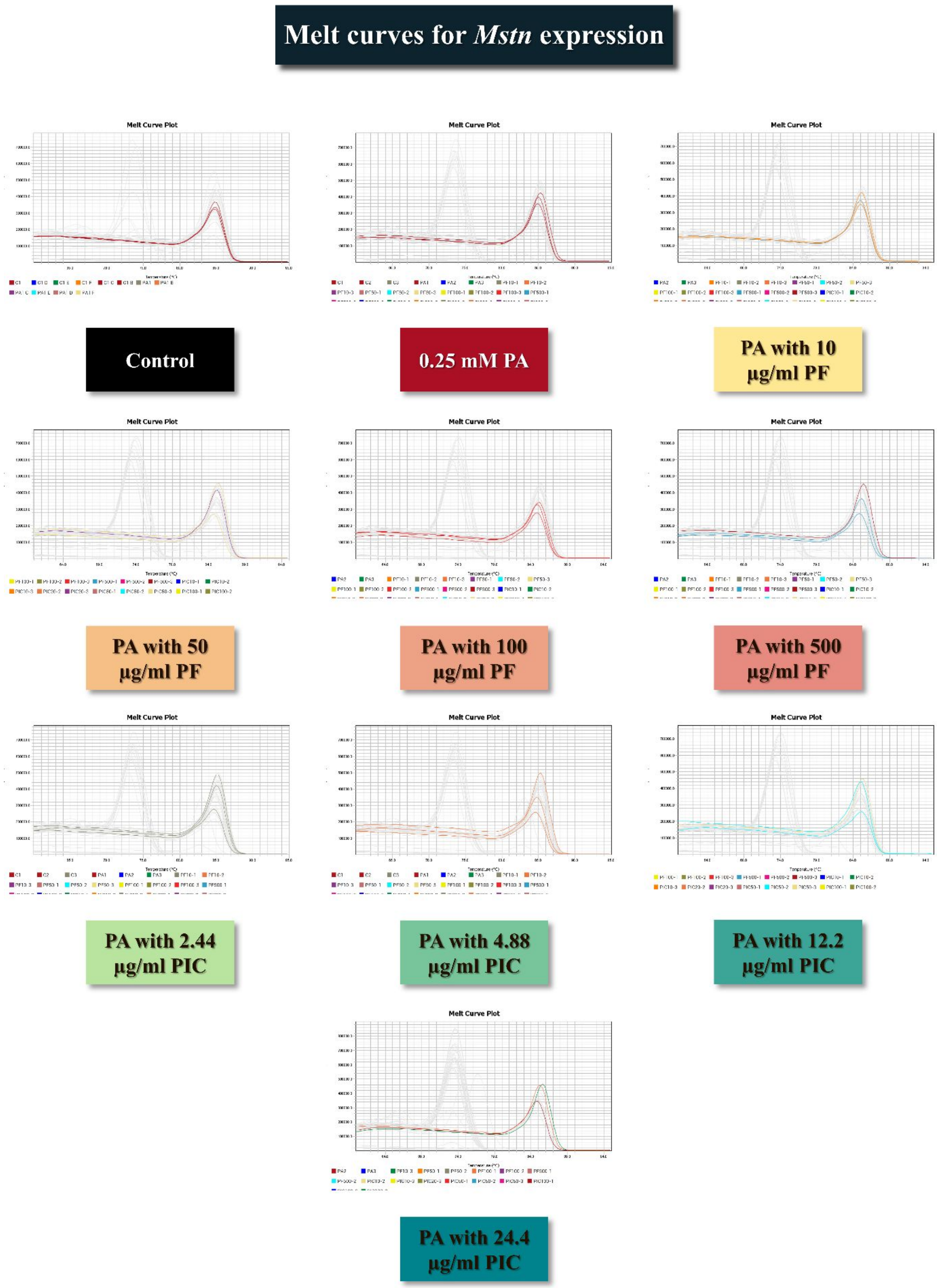

Figure S5. The melt curves of *Myh1*

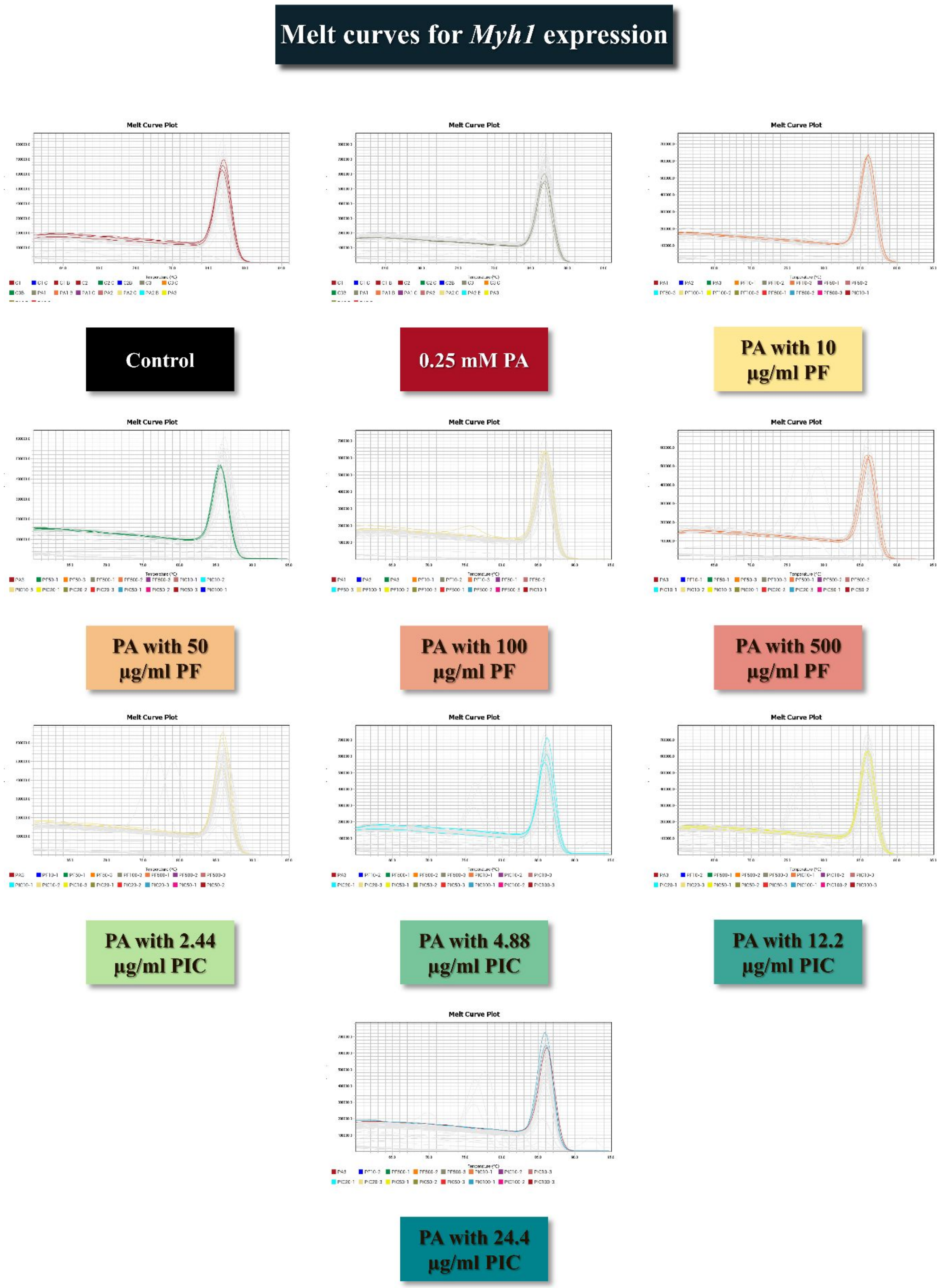

Figure S6. The melt curves of *Sirt1*

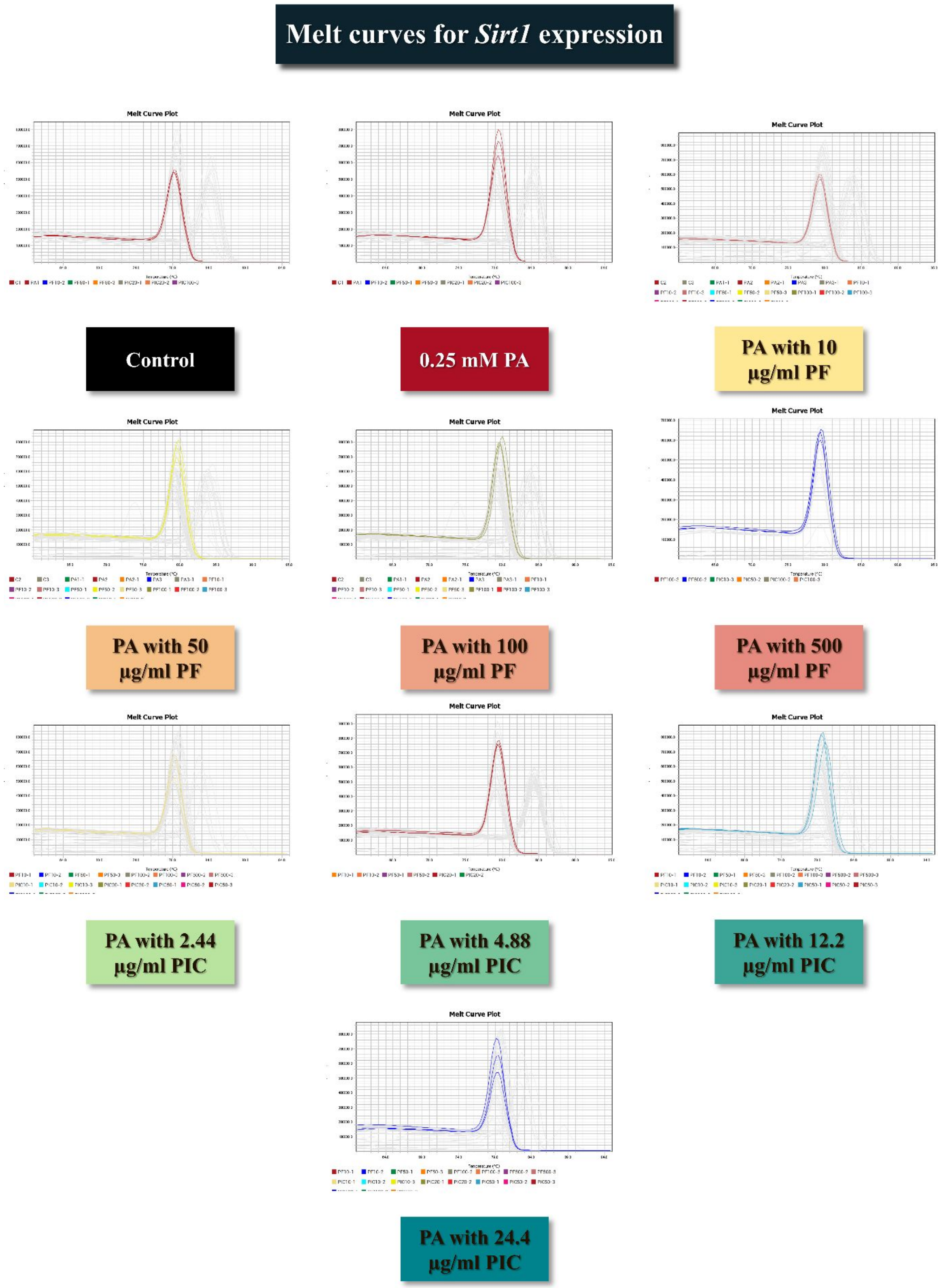

Figure S7. The melt curves of *Ucp3*

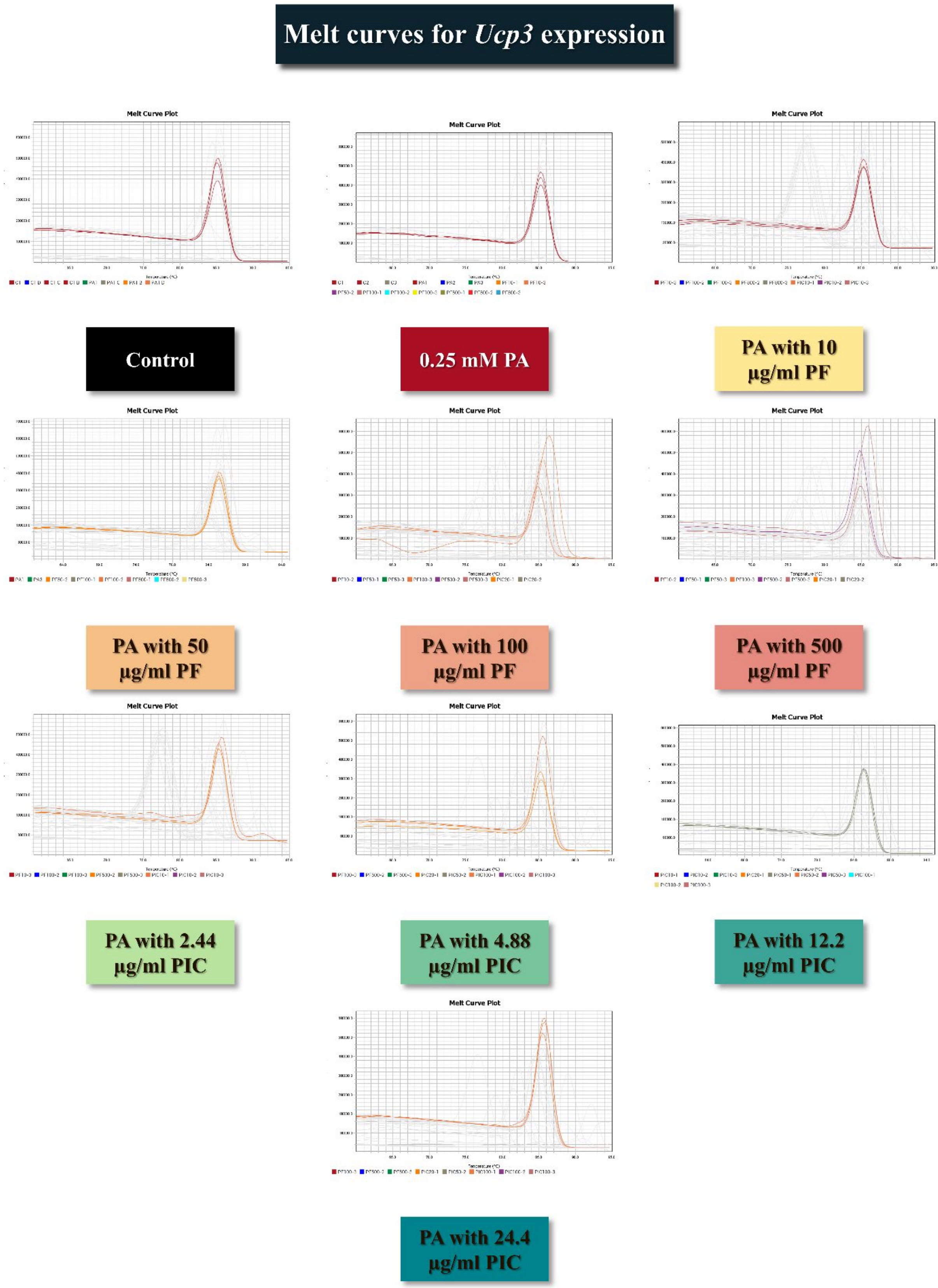

Table S1. Sequences of primers used for real-time PCR in this study.

Gene	Forward (5'-3')	Reverse (5'-3')	Annealing temperature
Dgat1	CCAGGTGGTGTCTCTGTTCC	GCTGGGAAGCAGAGAATGGT	59°C
Gapdh	AAATGGTGAAGGTCGGTGTG	GAATTTGCCGTGAGTGGAGT	57°C
Mstn	CTGTAACCTTCCCAGGACCA	TCTTTTGGGTGCGATAATCC	56°C
Myh1	CGGTCGAAGTTGCATCCCTA	TAGTTCCGCCTTCGGTCTTG	57 °C
Sirt1	AGGGAACCTTTGCCTCATCTAC	GGTGGCAACTCTGATAAATGAAC	54°C
Ucp3	GCCTACAGAACCATCGCCAG	GCCACCATCTTCAGCATACA	56 °C

Dgat1, diacylglycerol acyltransferase 1; *Gapdh*, glyceraldehyde-3-phosphate dehydrogenase; *Mstn*, myostatin; *Myh1*, myosin heavy chain 1; *Sirt1*, sirtuin 1; and *Ucp3*, uncoupling protein 3.
